# Incorporation of Natural Biostimulants in Biodegradable Mulch Films for Agricultural Applications: Ecotoxicological Evaluation

**DOI:** 10.3390/polym17223027

**Published:** 2025-11-14

**Authors:** Chelo Escrig Rondán, Celia Sevilla Gil, Pablo Sanz Fernández, Juan Francisco Ferrer Crespo, Cristina Furió Sanz

**Affiliations:** 1 Department of Agriculture and Aquatic Environment, AIMPLAS—Plastics Technology Centre, C. Gustave Eiffel n. 4, 46980 Paterna, Valencia, Spain; csevilla@aimplas.es (C.S.G.); psanz@aimplas.es (P.S.F.); 2 Microplastics Laboratory, AIMPLAS—Plastics Technology Centre, C. Gustave Eiffel n. 4, 46980 Paterna, Valencia, Spain; jfferrer@aimplas.es (J.F.F.C.); cfurio@aimplas.es (C.F.S.)

**Keywords:** plasticulture, mulch film, agriculture, biodegradation, natural biostimulants, sustainability, standard test, ecotoxicity

## Abstract

This study deals with the incorporation of biostimulants of natural origin in a biodegradable polymeric matrix, with the aim of developing mulch films that, when degraded in the soil, release bioactive compounds that improve soil quality and favor the agronomic growth of crops. Three types of commercial biostimulants were used: one based on seaweed extract, one on lignosulfonates, and one on plant-derived essential amino acids. To ensure the thermal stability of the biostimulant compounds during processing, thermogravimetric analyses (TGAs) were carried out, and a methodology based on the adsorption of the biostimulants onto porous substrates was developed, enabling their effective incorporation into the polymeric matrix. The formulations obtained have been processed by blown film extrusion at a pilot scale. In addition, the presence of film residues in soil was analyzed by pyrolysis–gas chromatography–mass spectrometry (Py-GC/MS). The results indicate that the proposed methodology supports the integrity of the biostimulants in the films obtained. After the incubation period studied, complete degradation of the biopolymer and the absence of film residues in the soil were confirmed. Furthermore, it was confirmed that this final product had no adverse effects on organisms that were representative of the two end-of-life scenarios, with the exception of the film functionalized with the commercial biostimulant based on seaweed extract, which showed a negative effect on terrestrial higher plants.

## 1. Introduction

The global production of plastics has experienced accelerated growth since their invention, owing to their advantageous properties and versatility for use in multiple sectors, with agriculture being one of the most significant. Agriculture Plastic & Environment Europe (APE Europe) estimated that in 2019 approximately 722 kilotonnes of plastics were placed on the European market for plasticulture applications, resulting in the generation of more than 1175 kilotonnes of agricultural plastic waste in Europe each year [1]. These enormous volumes of plastic waste have led to increasing environmental concern. Mulching films accounted for 76% of the total agricultural plastics marketed in 2019 [2]. The widespread use of conventional mulching films in agriculture has posed a significant challenge in terms of environmental impact and end-of-life management. After their removal from the field, these films are often contaminated with soil, plant residues, and agrochemicals, impurities that can account for between 30% and 80% of the total mass, making mechanical recycling technically and economically unfeasible without intensive cleaning operations [3,4,5]. Moreover, due to their low thickness and exposure to weathering, conventional films are prone to fragmentation and, consequently, to incomplete recovery, leaving plastic residues and microplastics (MPs) in the soil that can persist for years and negatively affect the agronomic quality of soils [6,7,8]. MPs on agricultural surfaces can be transported to aquatic systems through surface runoff during heavy rainfall events or by wind erosion processes, which may affect the sustainability of aquatic ecosystems [9,10].

In response to these environmental and logistical challenges, biodegradable plastic mulches (BDMs) have emerged as a more sustainable alternative. From an agronomic standpoint, several scientific studies have reported that BDMs provide performance comparable to that of polyethylene (PE) in multiple crops and contexts [11,12,13,14,15]. In parallel, life cycle assessments highlight that these BDMs are environmentally preferable to PE, as they eliminate the need for removal and cleaning operations and reduce the risk of plastic residues at the end of the cropping season [16,17,18]. Within this framework, mulch films are the only agricultural product for which biodegradability criteria have been established. Commission Delegated Regulation (EU) 2024/2787 stipulates that, in the case of soil, the main environment in which its useful life takes place, the plastic material must achieve at least 90% biodegradation within 24 months after the product’s functional period [19].

Furthermore, in the agricultural sector there is an additional concern related to the widespread use of chemical inputs as the most common practice in modern agriculture. Their intensive use has triggered a series of environmental and agronomic impacts that cast doubt on their sustainability in the medium and long term [20,21,22,23,24]. In response, the European Commission, within the framework of the Green Deal and the “Farm to Fork” strategy, has proposed to reduce nutrient losses from organic and mineral fertilizers by at least 50% by 2030, without compromising soil fertility, which would imply a reduction in fertilizer use of at least 20% [25]. Similarly, the EU has set a target to reduce the use and risk of chemical pesticides by 50% compared to the 2015–2017 average [26,27]. These strategic ambitions are framed within the new Common Agricultural Policy (CAP), which incorporates instruments such as eco-schemes to guide agricultural practices toward a more sustainable use of inputs [25].

In this context, natural biostimulants have emerged as a promising alternative and/or complement to improve nutrient use efficiency, enhance plant tolerance to abiotic stress conditions, and promote soil health, thereby contributing to reducing dependence on chemical inputs. Among these, seaweed extracts have shown average yield increases and physiological improvements in recent studies [28,29,30]. Complementarily, lignosulfonates act as chelating agents that enhance micronutrient availability and uptake and are associated with improved plant growth and quality [31,32,33,34]. Moreover, plant-derived amino acids have been reported in reviews and experimental studies to improve plant growth, nutrient use efficiency, and stress tolerance [35,36].

The incorporation of natural-origin biostimulants into BDMs not only paves the way for more sustainable agriculture but also integrates, within a single product, the physical crop control function with the delivery of beneficial bioactive compounds. Along the same research line, numerous studies have incorporated synthetic fertilizers into biodegradable polymers [37,38,39,40]. However, the reviewed literature has not yet reported biodegradable mulching films functionalized with natural biostimulants.

In this context, the main objective of the present study is to evaluate the technical and environmental feasibility of incorporating commercial natural-origin biostimulants into BDMs intended for agricultural applications. A strategy based on the adsorption of biostimulants onto porous substrates is employed to facilitate their incorporation into the polymer matrix and to mitigate their thermal degradation during processing. In addition, thermogravimetric analyses (TGAs) are carried out to characterize the thermal stability of the biostimulants and to ensure the preservation of their functional properties at compounding and extrusion temperatures. The study is complemented by ecotoxicity assays on representative organisms from aquatic (sea urchin larvae and microalgae) and terrestrial (higher plants and earthworms) ecosystems, with the aim of comparatively assessing the environmental safety of the films under different end-of-life scenarios. Finally, the presence of plastic residues in soils after film degradation is analyzed using advanced characterization techniques, including pyrolysis coupled with gas chromatography and mass spectrometry (Py-GC/MS), in order to determine the possible formation of microplastics and to confirm the complete biodegradation of the material.

## 2. Materials and Methods

### 2.1. Plastics and Additives

A commercial biodegradable soil biopolymer compliant with the EN 17033 standard [41] was used as the matrix for the preparation of masterbatches containing the biostimulants, followed by film extrusion. To improve the protection of the biostimulants against temperature and shear during the compounding and blown film extrusion processes, a porous-structured substrate with a narrow particle size distribution (average particle size d50 ≈ 13 μm) and a high absorption capacity of 270 mL DOA/100 g (ISO 19246) was used as carrier of the natural biostimulants [42]. Three commercial biostimulants of natural origin were included in the study: one based on seaweed extracts (BS1), another composed of essential amino acids (BS2), and a third formulated with lignosulfonates (BS3). All three biostimulants were in liquid form Table 1 shows the qualitative composition and format of the selected commercial biostimulants of natural origin.

### 2.2. Biological Material

Seeds of *Hordeum vulgare* and *Cucurbita maxima* were obtained from a company specialized in agricultural products. Prior to the ecotoxicity test, a germination test was carried out in Petri dishes to assess their viability. Germination capacity was tested in three replicates, and all plates reached the optimal germination rate, exceeding 70%, thus confirming the suitability of the seed batches. The seeds were kept in their sealed commercial packaging until use.

The *Eisenia fetida* worm colonies were obtained from a company specialized in vermiculture. The worms were kept in a phytotron, housed in trays with the vermicompost in which they were received (temperature 19 ± 4 °C, humidity 70–90%, neutral pH, darkness) until use. The worms were fed weekly with wheat flour and occasionally with fruit residues.

The microalga *Isochrysis galbana* was obtained from a stock maintained at a research center. The cultures were kept under controlled laboratory conditions, in filtered and sterilized seawater, under a continuous photoperiod (temperature 25 ± 2 °C, pH 8.1 ± 0.1). The diet consisted of a standard nutrient-based culture medium, F/2 Medium [43], routinely used.

Adult specimens of *Paracentrotus lividus* were collected from a wild population in the outer area of the Ría de Vigo (Galicia, NW of the Iberian Peninsula) between May and August. Approximately 100 individuals (50 males and 50 females) were housed in 150 L tanks under natural photoperiod conditions and running seawater (temperature 14 ± 2 °C, pH 8.1 ± 0.1) until use. The sea urchins were fed daily with green and brown algae, specifically *Ulva lactuca* and *Laminaria* sp.

### 2.3. Obtaining Functionalized Film

The porous substrates were loaded with the natural-origin biostimulants at a concentration of 50% (*w*/*w*). Prior to compounding, the selected commercial polymeric matrix, which meets biodegradation requirements under soil conditions, was dried in a dehumidifier at 60 °C for 4 h to reduce its moisture content. Subsequently, the substrates loaded with the biostimulants were incorporated into the biopolymer matrix at a concentration of 10% (*w*/*w*) through a compounding process. The mixture was processed using a PRISM Eurolab 16 XL (Thermo Scientific, Karlsruhe, Germany) co-rotating twin-screw extruder. The biopolymer matrix and the porous substrates loaded with the adsorbed biostimulants were fed through independent gravimetric feeders to ensure higher formulation accuracy. The extruded strand was continuously cut, and the resulting pellets were stored in sealed aluminum bags to prevent moisture absorption prior to further processing.

After obtaining the masterbatches of each natural biostimulant through compounding, they were incorporated into the biopolymer matrix up to a final concentration of 10% (*w*/*w*) and processed by blown film extrusion at pilot plant scale. The processing was carried out on a blown film extrusion line equipped with a single-screw extruder COLLIN E30E (Collin Lab & Pilot Solutions, Maitenbeth, Germany), a 30 mm diameter screw, a COLLIN Blasanlage BL 180/400 (Collin Lab & Pilot Solutions, Maitenbeth, Germany) blowing tower, and a 5 cm diameter die head. A conventional HDPE extrusion screw was used, with a compression ratio of 1:2. The screw speed was set at 40 rpm, and the temperature profile along the extruder was fixed between 130–150 °C (from feed to die). The blow-up ratio (BUR) was adjusted to 4:1 to achieve the required biaxial orientation. The films were produced under controlled temperature, pressure and draw ratio conditions, achieving a uniform final thickness of 25 µm for each formulation. Table 2 details the theoretical concentrations of the biostimulants throughout the different stages leading to the production of the functionalized films. The theoretical concentrations were calculated considering the proportions of biostimulant adsorbed onto the porous substrate and the subsequent dilutions in the polymer matrix during the compounding (masterbatch) and blown film extrusion stages.

### 2.4. Preparation of Samples for Ecotoxicity Assays and Evaluation of Mulching Film Residues by Py-GC/MS

Once the non-functionalized biopolymer film and the films functionalized with natural biostimulants were obtained, they were micronized using a ZM 200 Micronizer (Retsch GmbH, Haan, Germany) and sieved with an AS 300 Control Sieve Shaker (Retsch GmbH, Haan, Germany) to obtain a particle size range of 125–250 µm (ISO 10210 [44]).

The micronized non-functionalized mulching film was used as the sample for the ecotoxicity assays in aquatic environments (microalgae and sea urchin embryos). In parallel, all micronized films were incorporated into the soil substrate at 1% (*w*/*w*) (according to the EN 17033 standard) and subjected to a 90-day incubation period following the guidelines of ISO 20200 [45]. The different soil substrate samples obtained after the incubation period were used for the ecotoxicity assays in terrestrial environments (higher terrestrial plants and earthworms) and for the analysis of mulching film residues by Py-GC/MS.

### 2.5. Thermogravimetric Analysis (TGA)

In order to evaluate the thermal stability of the selected commercial biostimulants and ensure that the active compounds do not degrade during the different stages of processing, thermogravimetric analyses were performed for each of the biostimulants. The TGAs were performed using TGA5500IR equipment (TA Instruments, New Castle, DE, USA), in a temperature range of 30 °C to 900 °C, with a heating rate of 20 °C/min under an air atmosphere.

### 2.6. Procedures for Biological Testing

#### 2.6.1. Testing on Terrestrial Higher Plants

A seed germination test was carried out. Five milliliters of demineralized water were added to a Petri dish containing filter paper placed over a layer of cotton. Fifteen seeds of *Cucurbita maxima* and fifty seeds of *Hordeum vulgare* were placed on top of the filter paper, followed by a second filter paper covering the seeds. The plates were kept in darkness at room temperature. After five days, the number of germinated seeds was counted.

The terrestrial plant bioassays followed the procedures described in OECD 208 [46] and the modifications established in Annex B of UNE-EN 17033. Plant species from different families were used, selecting one monocotyledonous and one dicotyledonous species, in this case *Hordeum vulgare* and *Cucurbita maxima*, respectively. The test was carried out using the soil substrate obtained after incubation with the micronized films containing the incorporated biostimulants BS1, BS2, and BS3, since the active compounds embedded in the polymer matrix are released into the soil during use. Therefore, it is necessary to study the effect of these compounds in the terrestrial environment, in addition to that of the non-functionalized film.

The bioassay was conducted in pots containing 200 g of soil substrate prepared after the incubation period with the different micronized film samples. Twenty-five seeds of the same species were placed on the surface of each sample and covered with a thin layer of inert material (perlite). Four replicates were prepared for each sample. The pots were incubated in a phytotron under dark conditions at a temperature of 22 ± 5 °C and a relative humidity of 70 ± 5% during the germination period. After germination, the pots were exposed to the following conditions: photoperiod of 16 h light/8 h dark, temperature of 22 ± 5 °C, relative humidity of 70 ± 5%, and light intensity of 3300 lux. The test was concluded 15 days after 50% of the seeds had germinated in the control pots.

#### 2.6.2. Earthworm Test

The earthworm bioassays followed the procedures described in OECD 207 [47] and the modifications established in Annex C of the UNE-EN 17033, in accordance with ISO 11268-1 [48]. Earthworm species *Eisenia fetida* or *Eisenia andrei* were used as bioindicator organisms.

The bioassay was conducted in glass containers holding 500 g of soil substrate prepared after the incubation period with the different micronized film samples (BS2, BS3, and the non-functionalized biopolymer). Ten adult earthworms were added to each container, with a total of four replicates prepared for each sample. The containers were kept in a controlled chamber under the following conditions: continuous-light photoperiod, temperature of 20 ± 5 °C, relative humidity of 70 ± 5%, and light intensity of 3300 lux. The test was concluded after a 14-day incubation period.

#### 2.6.3. Microalgae Test

To carry out the bioassay, micronized non-functionalized biopolymer film was added at a concentration of 15 mg/L in filtered seawater, since it was considered that the biostimulants incorporated into the polymer matrix are released into the soil during the film’s service life, and that the plastic polymer samples reaching the aquatic environment would no longer contain the active compounds. These stock suspensions were kept under agitation for 24 h. One hour before performing the bioassays, each stock was sonicated, and the corresponding dilutions were prepared to obtain two concentrations: an environmental concentration of 0.0015 mg/L and a high concentration of 1.5 mg/L. These concentrations were defined internally by the research group specializing in marine pollution. The environmental concentration (0.0015 mg/L) represents realistic levels detected in coastal marine waters, where values in the range of 0.001–0.01 mg/L have been reported, especially in areas with moderate anthropogenic activity [49]. On the other hand, the high concentration (1.5 mg/L) is commonly used in ecotoxicological studies as an intensified exposure scenario, allowing the evaluation of sublethal effects and toxicity mechanisms under high stress conditions [50]. This experimental strategy allows the identification of dose-dependent responses and the characterization of sensitivity thresholds in early stages of marine organisms [51].

Microalgae bioassays were conducted in 10 mL vials, using four replicates per treatment. The vials were previously filled with either the high or environmental concentrations of the plastic sample. Two controls were included—one control contained vials with *Isochrysis galbana* in growth medium, and the other contained an aliquot of 100 µL of seawater (without the plastic sample)—to reproduce the exact conditions of the treatments and to rule out any potential effect of the seawater, used to prepare the plastic sample solutions, on growth.

Microalgae in the exponential growth phase were added to each vial (20,000 cells/mL) containing growth medium (L1 without silicates), followed by the addition of the appropriate aliquot of the plastic sample solution to reach the desired concentration. Microalgae fluorescence was measured at time zero (microplate reader—excitation and emission wavelengths of 450 nm and 650 nm, respectively). These measurements were repeated after 24 h, 48 h, and 72 h (T0, T1, T2, T3) of incubation in a growth chamber at 20 °C under constant illumination.

#### 2.6.4. Sea Urchin Embryo Test

To carry out the test, micronized non-functionalized biopolymer film was used, following the same procedure described for the microalgae test (Section 2.6.3). Embryonic development bioassays were conducted following the methods described by Saco-Álvarez et al. (2010) [52]. To minimize genetic variation, sea urchin gametes were obtained from a single pair of mature *Paracentrotus lividus* individuals per experiment. Fertilization was achieved by adding 10 μL of undiluted sperm to a dense suspension of oocytes in 40 mL of seawater. Fertilization success and oocyte density were evaluated by microscopic examination of three 25 μL aliquots. Fertilization rates consistently exceeded 99%, with a mean oocyte density of 7000 ± 200 oocytes/mL. Within 30 min after fertilization, the oocytes were transferred to 2.5 mL multiwell plates already containing the experimental treatments, at a concentration of 40 embryos/mL. Four replicates were prepared for each treatment. Incubation lasted 48 h at 20 °C in darkness without aeration. Subsequently, the samples were fixed with three drops of 40% buffered formalin and examined under an inverted microscope (Axiovert 40 CFL, Zeiss, Spain). Size measurements were obtained from images captured using a DFK 42BUC03 camera system (Imagingsource^®^, Bremen, Germany).

### 2.7. Procedure for Analyzing Mulching Film Residue

#### 2.7.1. Py-GC/MS Method

Py-GC/MS measurements were carried out using a micro-furnace pyrolyzer (EGA/Py-3030D, Frontier Laboratories Ltd., Koriyama, Fukushima, Japan.) equipped with an auto-shot sampler (AS-1020E, Frontier Laboratories, Ltd.). The pyrolyzer was interfaced directly to a split/splitless injection port of a GC instrument. Pyrolysis was performed by heating the sample rapidly to 600 °C in an inert helium atmosphere. The GC injection port was connected to a quadrupole mass detector through a separation column (Ultra ALLOY ± 5, 30 m × 0.25 mm i.d., coated with 0.5 μm film thickness of 5% diphenyl 95% dimethylpolysiloxane, Frontier Laboratories, Ltd.). The oven temperature is maintained at 40 °C for two minutes, then increased to 280 °C at a rate of 20 °C per minute and held for ten minutes. It is then increased to 320 °C at a rate of 40 °C per minute and held for fifteen minutes.

#### 2.7.2. Sample Preparation

The preparation of the soil substrate samples for analysis followed the procedure described in Section 2.4. The pretreatment consists of oxidizing the organic matter by hydrogen peroxide catalyzed with the Fenton reagent in previously oven-dried soil. Next, the plastic particles are separated from the matrix by density difference with salt, causing them to float to the surface. Sodium iodide (NaI) is then added until saturation, and the material is left for 12 h to facilitate separation. The supernatant fraction was filtered using QF1 quartz fiber filters. These were stored in an oven at 30 °C for at least three hours.

#### 2.7.3. Identification of Polymers

Pyrograms of the reference material, model polymer mixtures, and environmental samples were analyzed. The composition of each sample was determined by identifying the characteristic pyrolyzates of each polymer. Only one of the polymers present in the matrix is selected for semi-quantification. The compound terephthalic acid, di(but-3-enyl) ester, which is specific to the degradation of PBAT (poly(butylene) terephthalate), is present in the biopolymer matrix. It has therefore been selected as a characteristic compound for semi-quantifying the reduction in the biodegradable film released into the soil.

### 2.8. Statistical Analysis

A statistical analysis was performed to evaluate the effect of mulching film residues on the different bioindicator organisms. All analyses were conducted using the statistical software R version 4.5.0 (R Core Team, Vienna, Austria).

An analysis of variance (ANOVA) was performed to determine whether significant differences existed among the different treatments, followed by Tukey’s post hoc test to identify in greater detail which treatments showed those differences.

## 3. Results

### 3.1. Thermogravimetric Analysis (TGA)

**Figure 1:**
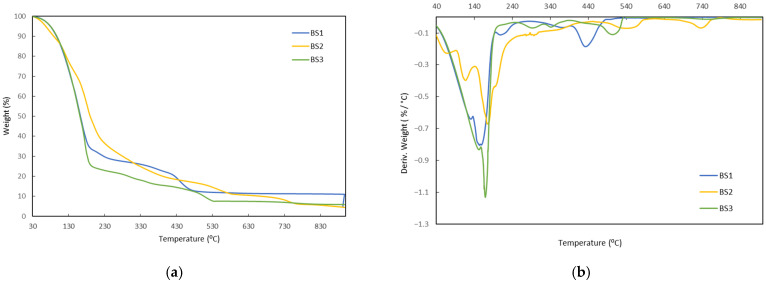
Mass loss curves as a function of temperature (**a**) and their derivative (**b**) obtained from thermogravimetric analysis (TGA) tests of selected natural biostimulants (BS1, BS2, and BS3).

The thermogravimetric curves of the biostimulants (a) BS1, BS2, and BS3 and their derivative (b) are shown in Figure 1. In the cases of BS1 and BS3, an initial mass loss with a very similar profile is observed in the range 30–180 °C, with maxima in the DTG curve at 183 °C and 170 °C, respectively. In the case of BS2, three distinct peaks that overlap slightly are observed in the derivative curve of the thermogram. This initial mass loss represents between 65 and 75% of the total mass of the biostimulants.

Next, the three products exhibit different degradation profiles. BS1 shows two main stages of mass loss: one between 180–320 °C, with a maximum in the DTG curve at 210 °C, and another between 360–520 °C, with a maximum at 430 °C. The final residue is 11%. BS2 shows a continuous decrease in mass from 180 °C to 450 °C, with no pronounced peaks in the DTG curve. Subsequently, a more pronounced loss is observed between 450 and 650 °C, with a maximum degradation rate around 440 °C, followed by a final stage between 650 and 800 °C, with a peak at 760 °C. The final residue is 4.5%. In the case of BS3, the mass loss occurs progressively between 180 and 400 °C, with two overlapping peaks in the DTG curve at 290 °C and 345 °C. The last significant stage is observed between 400 and 530 °C, with a maximum at 505 °C, resulting in a final residue of 6%.

These differences in profiles reflect the different composition of the biostimulants and the presence of inorganic components. The interpretation of these results and their relationship to the stability of the active compounds during processing is detailed in the Discussion (Section 4).

### 3.2. Biological Tests

#### 3.2.1. Germination Rate and Plant Biomass

**Figure 2:**
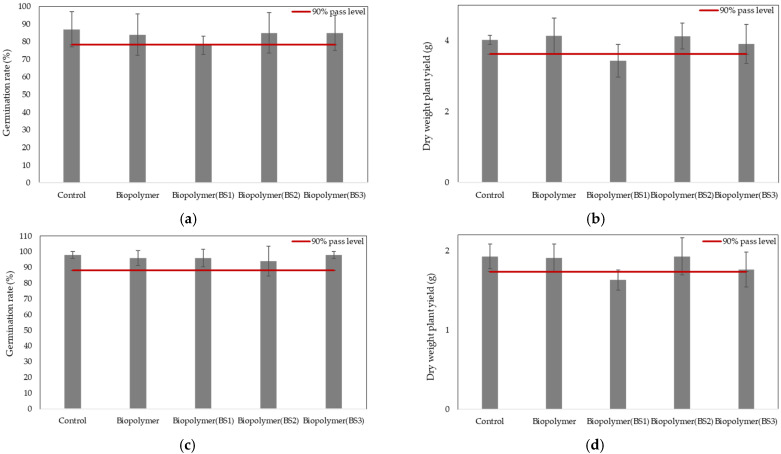
Bar chart showing germination rate (%) and plant yield in dry weight (g) for the different treatments in the terrestrial higher plants tested: *Cucurbita maxima* (**a**,**b**) and *Hordeum vulgare* (**c**,**d**). The red line represents 90% of control. ANOVA (**a**) *p* = 0.757, (**b**) *p* = 0.176, (**c**) *p* = 0.832, (**d**) *p* = 0.149.

The samples tested did not show any ecotoxicological effects on any of the plant species, as germination and biomass in the soil exposed to the different plastic samples exceeded 90% of the corresponding white soil, complying with the criteria established in the EN 17033 standard, with the exception of the functionalized biopolymer with BS1, which was therefore discarded for the rest of the tests (Figure 2). The differences between germination and biomass between the treatments and the control were not statistically significant (one-way ANOVA). The results of Tukey’s post hoc test confirmed the absence of significant differences. The numerical data corresponding to Figure 2 are provided in Table A1, Table A2, Table A3 and Table A4, to which the reader is referred for further details.

#### 3.2.2. Mortality Rate and Body Mass of Earthworms

**Figure 3:**
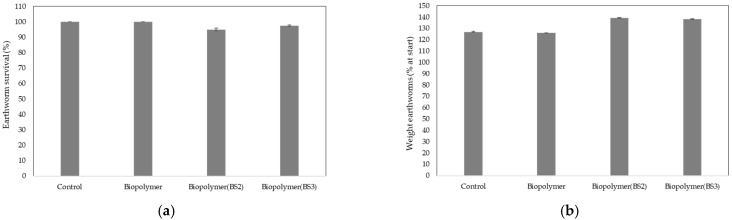
Bar chart showing mortality rates (%) (**a**) and body mass (% at start) (**b**) in relation to the different treatments in *Eisenia fetida*. ANOVA (**a**) *p* = 0.552, (**b**) *p* = 0.002.

The samples tested did not show any ecotoxicological effects on invertebrates, as mortality and biomass in the soil exposed to the different plastic samples was less than 10% compared to the control, complying with the criteria established in the EN 17033 standard (Figure 3). After performing the one-way ANOVA analysis, no significant differences in mortality were observed. However, significant differences in body mass were observed. Using Tukey’s post hoc test, it was identified that this significance was due to specific differences between treatments. The numerical data corresponding to Figure 3 are provided in Table A5, to which the reader is referred for further details.

#### 3.2.3. Growth Rate in Microalgae

**Figure 4:**
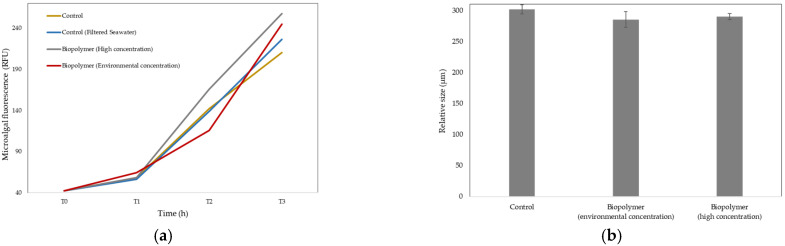
Line diagram showing fluorescence (RFU) at 0, 24, 48, and 72 h for the different treatments in *Isochrysis galbana* (**a**). Bar chart showing relative size (µm) versus different treatments at 0 h in *Paracentrotus lividus* (**b**). ANOVA (**a**) *p* = 0.046, (**b**) *p* = 0.184.

Exposure to the two tested concentrations of the plastic sample (0.0015 mg/L and 1.5 mg/L) did not produce inhibitory effects compared to the controls, following a similar upward trend in all treatments. The absence of a significant reduction in fluorescence compared to the controls indicated that no adverse effect occurred (Figure 4a). Although the effect of treatment was not significant, the interaction with the time variable was significant (two-way ANOVA). The significance of the interaction indicates that the effect of treatments on growth rate is time-dependent. The results of Tukey’s post hoc test confirmed these significant differences. The numerical data corresponding to Figure 4a are provided in Table A6, to which the reader is referred for further details.

#### 3.2.4. Growth Rate in Sea Urchin Embryos

The results showed some discrepancy between the two concentrations tested in the plastic sample, as the environmental concentration (0.0015 mg/L) showed a slightly greater reduction in larval size than the high concentration of plastic particles (1.5 mg/L). (Figure 4b). Despite this, these results suggest that the plastic sample did not present an obvious acute ecotoxicological risk to larval development under the experimental conditions tested, since the magnitude of the effect is low and does not differ significantly from the control (one-way ANOVA). The numerical data corresponding to Figure 4b are provided in Table A7, to which the reader is referred for further details.

### 3.3. Study of Degradation Compounds in Mulch Films

**Figure A1:**
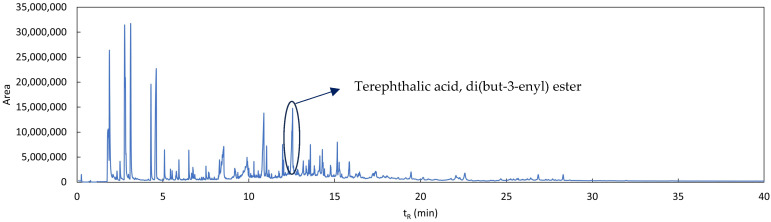
Chromatogram corresponding to the sample at the start of the test.

**Figure A2:**
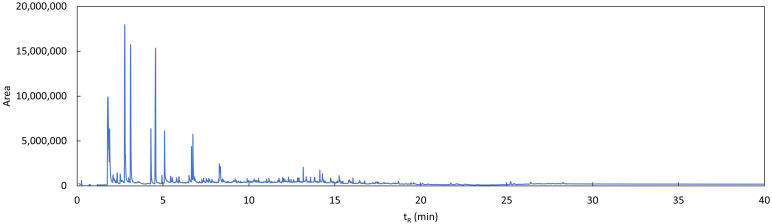
Chromatogram corresponding to the sample after 60 days of starting the test.

**Figure A3:**
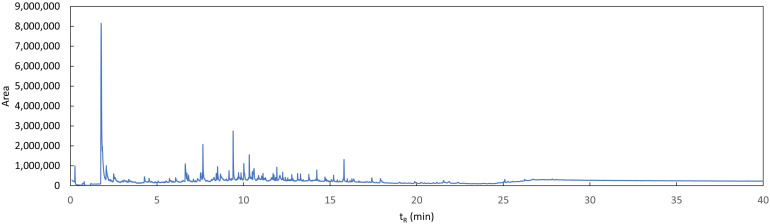
Chromatogram corresponding to the sample after 120 days of starting the test.

To verify the quantitative performance of the analysis of mulching film residues using Py-GC/MS, calibration curves were made using the reference materials. The calibrations curves were linear (correlation coefficients: r ≥ 0.985), representing the area of the characteristic compound against different biopolymer concentrations. The analyte has a detection limit (LOD) and a quantification limit (LOQ) of 0.05 mg and 0.17 mg, respectively. The presence of film residues is evaluated at the beginning and every 60 days. As shown in Table 3, immediately after incubation begins, a concentration of 0.004 ± 0.002% biopolymer is obtained in the sample. After 60 and 120 days, the biopolymer is below the LOD. The amount of organic particles in the film is gradually reduced until there are no residues left at the end of the test. Figure A1, Figure A2 and Figure A3 illustrate this reduction.

## 4. Discussion

TGA were performed on the commercial natural-origin biostimulants in order to study the potential thermal degradation of the different biostimulant active compounds during the compounding and blown film extrusion processes.

As shown in Figure 1, for all biostimulants the greatest mass loss (65–75%) observed in the TGA curves occurs within the temperature range of 30–180 °C. In the cases of BS1 and BS3, an initial mass loss with a very similar profile is observed, which can be attributed to the removal of the aqueous phase of the biostimulants, bound water, and other possible solvents. In the case of BS2, the three slightly overlapping differentiated peaks, in addition to the loss of the aqueous phase, may be associated with the presence of other solvents with lower evaporation points [53] with other volatile compounds or ammonium salts [54] present in the formulation.

In the case of BS1 and BS2, the second stage (180–360 °C) corresponds to the degradation of plant-derived amino acids (Table 1) through decarboxylation and deamination, with the release of CO_2_ and NH_3_, as previously described by TGA–MS analyses on model amino acids [55] or BS1, this interval may also be related to the depolymerization of polysaccharides present in seaweed extracts, whose thermal decomposition typically occurs within the 200–300 °C range [56,57]. In the case of BS3, the peaks observed in the DTG curve within this range can be associated with the main depolymerization and rearrangement reactions of lignosulfonates [58,59,60]. The peaks corresponding to the third stage (360–650 °C) are associated with the continued breakdown of the more resistant fractions of polysaccharides, as well as aromatic and phenolic compounds present in seaweed extracts and lignosulfonates, and with the progressive consumption of char promoted by the catalytic action of metallic micronutrients present in the formulations [60,61,62]. The late peak of BS2, showing a DTG maximum at 760 °C, is consistent with the final oxidation of a highly refractory char generated in previous stages. In humic substances analyzed by TG–FTIR/DSC in air, an intensification of CO_2_ release and exothermic events between approximately 650–850 °C has been observed, indicating the consumption of highly condensed carbon [63].

Overall, the TGA profiles showed that the main mass-loss degradation events of the biostimulants start above 180–200 °C. Since the processing of the biopolymer matrix takes place at 130–150 °C in twin-screw extruders, these data support that no major thermal degradation processes occur for the biostimulants within the processing window, beyond the expected release of water and low-boiling volatiles. However, TGA cannot exclude minor chemical rearrangements or subtle changes in specific functional groups that do not involve significant mass loss.

Internationally recognized ecotoxicological methods for soil organisms recommend the use of higher plants and earthworms as bioindicators of ecological disturbances, whereas for aquatic organisms, the use of microalgae and sea urchin embryos is recommended.

Higher plants are commonly used as biological models in ecotoxicity bioassays. In this study, *Hordeum vulgare* and *Cucurbita maxima* were selected due to their rapid growth, sensitivity to environmental stress factors, and agricultural relevance. The concentration of the plastic sample tested was 1%, which is considerably higher than the expected loading of a biodegradable mulching film in soil, as indicated in the EN 17033 standard. According to the criteria established in UNE-EN 17033 to ensure the absence of toxicity, both germination rate and biomass must exceed 90% of those observed in the control.

As shown in Figure 2, the results obtained in these bioassays indicated that exposure to the different treatments, except for BS1, did not produce adverse effects on the early development and growth of either plant species. The results of the present study differ from previous findings, such as those reported by Wang et al. (2024) [64] where a significant reduction in lettuce growth was observed. These findings also contrast with other studies [65] that reported adverse effects at higher concentrations, up to 10% of plastic sample, which are not representative of real environmental conditions. The plastic sample functionalized with BS1 showed an adverse effect on both terrestrial plants. This may be attributed to the fact that exposure to high concentrations of the biostimulant can induce phytotoxicity [66,67]. For this reason, this functionalized plastic sample was excluded from the remaining tests, suggesting that seaweed extracts should be used with caution as biostimulants.

The use of earthworms is considered a standard ecotoxicological test, as these organisms live in permanent contact with soil, are widely distributed, play key ecological roles, reproduce rapidly, and are easily maintained under laboratory conditions. In this study, *Eisenia fetida* was selected as the test species, as it is recommended for this type of assay. The concentration of the plastic sample tested was 1%. According to the criteria established in UNE-EN 17033 to ensure the absence of toxicity, the difference in mortality and biomass of adult earthworms between the soil exposed to the test material and the corresponding unexposed control soil must be less than 10% of that of the control soil. As shown in Figure 3, the results obtained in these bioassays demonstrated that exposure to the different treatments did not produce adverse effects on earthworm survival or body mass. The results of the present study are consistent with the findings of previous studies [68,69,70] in which earthworms were used as bioindicator organisms for these types of plastic residues. The increase in body mass observed in earthworms in treatments BS2 and BS3 could be attributed to an indirect fertilizing effect, because biostimulants improve nutrient availability and soil structural quality, which can lead to increased consumption and weight gain in earthworms. These results are consistent with the findings of experimental studies [71] showing increases in earthworm weight when organic amendments are added or during vermicomposting processes with nutritious substrates.

Microalgae, as phytoplankton of great importance in aquatic ecosystems, have been widely selected as model organisms for ecotoxicological testing. In this study, the marine microalga *Isochrysis galbana* was selected to investigate the potential effects of bioplastic residues. Chlorophyll a fluorescence was measured, as this parameter is related to the growth and photosynthetic activity of these organisms. As shown in Figure 4a, the results indicated that exposure to the two concentrations of the plastic sample (0.0015 mg/L and 1.5 mg/L) did not produce adverse effects, showing a similar trend across all treatments. The absence of a significant reduction in fluorescence compared with the controls indicated that no detrimental effect occurred in this aquatic organism. This finding is consistent with the study conducted by Xu et al. (2024) [72] in which no significant inhibition of fluorescence was observed after exposure of *Chlorella pyrenoidosa* to biodegradable plastic residues. Conversely, the results do not agree with previous reference studies [73] that used concentrations higher than those established in the present study, which are not considered environmentally realistic. Nevertheless, further studies are required to confirm these trends and establish conclusive assessments of ecotoxicological risks in the microalga.

Embryonic and larval bioassays using marine invertebrates have been frequently employed to assess the toxicity of certain substances. Among the most commonly used embryo–larval bioassays are those performed with sea urchins, and *Paracentrotus lividus* was selected for this study. As shown in Figure 4b, the results obtained from the sea urchin embryo test (SET) indicated that exposure to the tested concentrations of the plastic sample caused a slight reduction in larval size compared with the control. At the same time, an inconsistency was observed between the two tested concentrations, as the environmental concentration (0.0015 mg/L) resulted in a slightly greater reduction in larval size than the higher concentration of the plastic sample (1.5 mg/L). Given the low magnitude of the effect, the lack of significant differences from the control, and the absence of a clear concentration–response relationship, the results suggest no evident acute ecotoxicological risk under the experimental conditions tested. These results contradict previous studies [74,75] that reported toxicity of biodegradable polymers in marine invertebrates; however, further research is required to more thoroughly evaluate the potential ecotoxicological effects of these plastic residues at a comparable level.

To study the film degradation, terephthalic acid, di(but-3-enyl) ester—present in the biopolymer matrix—was used as a characteristic compound [76]. A sample was taken at the beginning of the experiment, and the analysis showed a biopolymer concentration of 0.004 ± 0.002%. Additional samples were collected after 60 and 120 days from the start of incubation, and the biopolymer residue in the samples was found to be below the limit of detection (LOD). This indicates that the biopolymer begins to degrade, and after 60 days of incubation, no residues of the material were detected in the soil samples.

## 5. Conclusions

The conclusions of the present article can be summarized in three key points:

1. The processing results demonstrated that it was possible to incorporate the commercial biostimulants into the biopolymer matrix through stable and reproducible operations, with no issues during twin-screw extrusion and no exudation of the biostimulant in the blown film. The TGA profiles showed that the main degradation events involving significant mass loss of the biostimulants start above 180–200 °C, whereas matrix processing takes place within the 130–150 °C range. Therefore, these data support that no major thermal degradation of the biostimulants occurs during compounding and extrusion, beyond the expected release of water and low-boiling volatiles or the occurrence of minor chemical modifications that do not involve detectable mass loss. Overall, the results confirm the feasibility of producing biodegradable films functionalized with natural biostimulants without compromising their processability.
2. The results of the ecotoxicological tests in terrestrial environments demonstrated that exposure of higher plants to the residues of the functionalized biodegradable mulching films did not produce any adverse effects, except for the film functionalized with BS1, which was therefore excluded from this application. Regarding the earthworm bioassays, no acute adverse effects were observed. In aquatic environments, the results obtained from the microalgae fluorescence study showed no adverse effects of the unfunctionalized film residue on this aquatic organism. Likewise, the results from the sea urchin larval bioassay suggested no evident acute ecotoxicological risk under the experimental conditions tested. This research indicates that the residues from the plastic samples derived from biodegradable mulching films intended for agricultural use did not show adverse effects on organisms that are representative of the two end-of-life scenarios for this plasticulture product. Additionally, the findings of this study highlight the need for long-term studies to assess potential chronic effects and further research to enable a comprehensive risk evaluation; for example, based on studies of the evolution of active compounds in soil, it would be interesting to conduct ecotoxicological tests in aquatic environments to assess the potential environmental impact of natural biostimulants.
3. To evaluate the presence of residues at the end of incubation, soil substrate samples were prepared with simulated plastic residue at a proportion of 1% (according to UNE-EN 17033), which had previously been micronized. The soil sample containing the micronized film was subjected to an incubation period of 120 days. The results showed that, at the beginning of the experiment, the biopolymer was present at a concentration of 0.004 ± 0.002% in the sample. After 60 and 120 days, the analysis was repeated, and the biopolymer concentration was found to be below the LOD. This result indicates that the biopolymer undergoes very rapid degradation in the terrestrial environment and that, after 60 days, no film residues were detected.

It should be noted that this study did not aim to evaluate the biostimulant effect of the functionalized films on higher plants during the crop cycle. Looking ahead, field agronomic studies would be of interest to assess the functional viability and the potential biostimulant effect of the mulching films developed in this work, and to investigate how the active compounds evolve in the soil environment not only during the service life of the films, but particularly as biodegradation progresses. It should be noted that the functional concept of this system is not based on controlled release during use, but rather on the delivery of bioactive molecules upon biodegradation in soil. In this context, assessing the temporal evolution of the presence and availability of these compounds under realistic cultivation conditions would provide a valuable direction for future work, complementing the present demonstration of technical feasibility, processability, and environmental safety.

## Figures and Tables

**Table 1:** Qualitative composition of selected commercial biostimulants.

Biostimulant	Major Component	Minor Component
BS1	Seaweed extract	Plant amino acids Complexed iron
BS2	Plant amino acids	Organic acids of plant origin
BS3	Lignosulfonates	Micronutrients Organic nitrogen

**Table 2:** Theoretical concentrations of biostimulants during the different stages until the functionalized films are obtained.

In Porous Substrate (% *w*/*w*) *	In Masterbatch (% *w*/*w*)	In Final Film (% *w*/*w*)
50	5	0.5

* Biostimulant relative to the total mass of the porous substrate with the biostimulant.

**Table 3:** Biopolymer concentration relative to the duration of the experiment.

Experiment Time (Day)	Biopolymer Concentration ± s (%)
0	0.004 ± 0.002
60	<LOD
120	<LOD

## Data Availability

The original contributions presented in this study are included in the article. Further inquiries can be directed to the corresponding author.

## Abbreviations

The following abbreviations are used in this manuscript:

%	Percentage
°C	Degrees Celsius
µL	Microliter
µm	Micrometer
ANOVA	Analysis of Variance
APE	Agriculture Plastic Environment
AVG	Average
BDMs	Biodegradable Plastic Mulches
BS1	Biostimulant based on algae extracts
BS2	Biostimulant based on essential amino acids
BS3	Biostimulant based on lignosulfonates
cm	Centimeter
CO_2_	Carbon Dioxide
CAP	Common Agricultural Policy
DOA	Dibutyl phthalate Oil Absorption
DTG	Derivative Thermogravimetry
EU	European Union
g	Gram
GC	Gas Chromatography
h	Hour
i.d.	Interior Diameter
ISO	International Organization for Standardization
L	Liter
L/D	Length–Diameter
LOD	Detection Limit
LOQ	Quantification Limit
Ltd.	Limited
m	Meter
mg	Milligram
min	Minutes
mL	Milliliter
mm	Millimeter
MPs	Microplastics
NaI	Sodium Iodide
NH_3_	Ammonia
NW	Northwest
nm	Nanometer
Ø	Diameter
OECD	Organisation for Economic Co-operation and Development
PBAT	Poly(butylene) terephthalate
PE	Polyethylene
Py-GC/MS	Pyrolysis–Gas Chromatography–Mass Spectrometry
RFU	Relative Fluorescence Unit
SD	Standard Deviation
SET	Sea Urchin Embryo Test
TGA	Thermogravimetric Analysis
TGA-MS	Thermogravimetric Analysis coupled with Mass Spectrometry
TG-FTIR/DSC	Thermogravimetry coupled with Fourier Transform Infrared Spectroscopy and Differential Scanning Calorimetry
*w*/*w*	Weight-to-weight

## Appendix A

**Table A1:** Germination rate of *Cucurbita maxima*.

Test Series	Germination Rate (%)	
	AVG	SD
Control	87.00	10.00
Biopolymer	84.00	11.78
Biopolymer with BS1	78.00	5.20
Biopolymer with BS2	85.00	11.50
Biopolymer with BS3	85.00	10.00

With AVG = average and SD = standard deviation.

**Table A2:** Absolute dry weight yield of *Cucurbita maxima*.

Test Series	Dry Weight Yield (g)	
	AVG	SD
Control	4.01	0.12
Biopolymer	4.13	0.49
Biopolymer with BS1	3.43	0.46
Biopolymer with BS2	4.12	0.37
Biopolymer with BS3	3.90	0.55

With AVG = average and SD = standard deviation.

**Table A3:** Germination rate of *Hordeum vulgare*.

Test Series	Germination Rate (%)	
	AVG	SD
Control	98.00	2.31
Biopolymer	96.00	4.62
Biopolymer with BS1	96.00	5.70
Biopolymer with BS2	94.00	9.50
Biopolymer with BS3	98.00	2.30

With AVG = average and SD = standard deviation.

**Table A4:** Absolute dry weight yield of *Hordeum vulgare*.

Test Series	Dry Weight Yield (g)	
	AVG	SD
Control	1.93	0.15
Biopolymer	1.91	0.17
Biopolymer with BS1	1.63	0.12
Biopolymer with BS2	1.93	0.23
Biopolymer with BS3	1.76	0.22

With AVG = average and SD = standard deviation.

**Table A5:** Survival, mortality and live weight yield of *Eisenia fetida*.

Test Series	Survival (%)		Mortality (%)	Live Weight Yield			
				(g per Worm)		(% of Start)	
	AVG	SD	AVG	AVG	SD	AVG	SD
Control	100.00	0	0	0.34	0.01	126.92	0.59
Biopolymer	100.00	0	0	0.38	0.03	125.99	0.22
Biopolymer with BS2	95.00	1.00	5.00	0.35	0.01	139.17	0.42
Biopolymer with BS3	97.50	0.50	2.50	0.31	0.01	138.15	0.22

With AVG = average and SD = standard deviation.

**Table A6:** Fluorescence of *Isochrysis galbana*.

Test Series	Microalgal Fluorescence (RFU)			
	T0	T1	T2	T3
Control	42.30	57.80	142.20	210.20
Control (Filtered Seawater)	42.30	56.20	139.00	226.20
Biopolymer (High concentration)	42.30	58.25	165.75	257.50
Biopolymer (Environmental concentration)	42.30	64.25	115.75	244.75

T = time.

**Table A7:** Growth rate of *Paracentrotus lividus*.

Test Series	Relative Size (µm)	
	AVG (T0)	SD
Control	301.60	7.34
Biopolymer (High concentration)	285.34	12.55
Biopolymer (Environmental concentration)	290.19	4.91

With AVG = average and SD = standard deviation.
